# Detection of Virulence and β-lactamase resistance genes of non-typhoidal Salmonella isolates from human and animal origin in Egypt "one health concern"

**DOI:** 10.1186/s13099-023-00542-3

**Published:** 2023-03-30

**Authors:** Mohamed S. Diab, Asmaa S. Thabet, Mohamed Abd Elsalam, Rania M. Ewida, Sotohy A. Sotohy

**Affiliations:** 1 Department of Animal Hygiene and Zoonoses, Faculty of Veterinary Medicine, New Valley University, El-Kharga, Egypt; 2 Assiut Lab., Animal Health Research Institute, ARC, Asyut, Egypt; 3 Department of Food Hygiene (Milk Hygiene), Faculty of Veterinary Medicine, New Valley University, El-Kharga, Egypt; 4 Department of Animal, Poultry and Environmental Hygiene, Faculty of Veterinary Medicine, Assiut University, Asyut, Egypt

**Keywords:** Antibiotic, Genes, Human, Milk, Resistance, Virulence, Zoonosis

## Abstract

**Background:**

Non-typhoidal *Salmonella* (NTS) is a major foodborne zoonotic pathogen worldwide. In the current study, Various NTS strains were isolated from (cows, milk and dairy products in addition to humans) in New Valley and Assiut Governorate, Egypt. NTS were firstly serotyped and tested by antibiotic sensitivity test. Secondly, some virulence genes and Antibiotic resistance genes have been identified by using PCR. Finally, Phylogenesis was performed depending on the *invA* gene, for two S. typhimurium isolates (one of animal origin and the other of human origin for evaluating zoonotic potential).

**Results:**

Out of 800 examined samples, the total number of isolates was 87 (10.88%), which were classified into 13 serotypes, with the most prevalent being *S. Typhimurium* and *S. enteritidis*. Both bovine and human isolates showed the highest resistance to clindamycin and streptomycin, with 90.80% of the tested isolates exhibiting MDR. The occurrence of the *invA* gene was 100%, while 72.22%, 30.56%, and 94.44% of the examined strains were positive for *stn*, *spvC*, and *hilA* genes, respectively. Additionally, *blaOXA-2* was detected in 16.67% (6/ 36) of the tested isolates, while *blaCMY-1* was detected in 30.56% (11of 36) of the tested isolates. Phylogenesis revealed a high degree of similarity between the two isolates.

**Conclusions:**

The high occurrence of MDR strains of NTS in both human and animal samples with high degree of genetic similarity, shows that cows, milk and milk product may be a valuable source of human infection with NTS and interfere with treatment procedures.

## Background

NTS is a major public health concern worldwide. *Salmonella* infections results in 93.8 million cases of gastroenteritis and 155,000 deaths annually [1]. Diarrhea, abdominal pain, and vomiting are the main characteristics of self-limiting gastroenteritis caused by NTS in individuals of all ages. However, severe invasive disease with complicated extraintestinal illness, bacteremia, and meningitis can be observed in children, the elderly, and immunocompromised patients [2, 3].

The main sources of human salmonellosis are contaminated food products of animal origin such as dairy products and direct contact with infected animals [4, 5]. *Salmonella* infection on cattle farms continues to be a major problem, causing economic losses and increasing the risk of transmission to humans. NTS shedding occurs in the feces of sick, recovered, and asymptomatic cattle, and the organism can survive for a long time in a favorable environment outside the host [6]. The presence of NTS in animal feces can increase intra-herd transmission, accidental spread to other herds, environmental contamination, and risk of human infection [7].

Many virulence genes have been linked to the pathogenicity of *Salmonella*, and the severity of infection depends mainly on the presence or absence of the *invA*, *stn*, *hila*, and *spvC* genes. These virulence genes are located either chromosomally or plasmidically and encodes products that help NTS interaction with the host cells. [8, 9]. *The invA* gene is a biomarker for NTS detection and play major role in invasion of epithelial cells. Whereas, *stn* gene encode for an enterotoxin production and *spvC* encodes for systemic infection in human and replication in animal [10–12].

The therapeutic and non-therapeutic use of various antibiotics in food-producing animals and their misuse in humans have led to the emergence of antimicrobial-resistant NTS. Consequently, the incidence of MDR *Salmonella* has increased in the recent decades. [13]. Plasmid genes can transfer antimicrobial resistance between bacteria, which can lead to the emergence of MDR strains. The global effort aims to limits antibiotic-resistant strains, as infection leads to a significant foodborne hazard to human [14–16].

Therefore, information on the *Salmonella* serotypes circulating in a given geographic area, virulence genes, and antimicrobial resistance is essential. The objectives of this study were to investigate the occurrence, distribution of different serotypes, virulence genes and antibiotic resistance of NTS in Assiut and New Valley Governorates.

## Results

A total of 800 samples from different sources including raw milk, kareish cheese, Damietta cheese, yoghurt, ice cream, animal fecal swabs, human fecal swabs and hand swabs were examined for *Salmonella*. Out of the examined samples, 10.88% (87) were positive for *Salmonella* by conventional methods. These isolates were serotyped using "O" and "H" antisera and the results showed 13 different *Salmonella* serotypes including S. typhimurium, S. enteritidis, S. tsieve, S. infantis, S. larochelle, S. virchow, S. molade, S. haifa, S. shubra, S. alfort, S. essen, S. apeyeme and S. heidelberg (Tables 1 and 4).

**Table 1:** Overall occurrence of NTS

Samples	Positive	%
Animal origin (n = 560)	67	11.96
Human origin (n = 240)	20	8.33
Total	87	10.88

An overall 87 *Salmonella* isolates tested for antibiotic susceptibility for 14 different antibiotics as shown in (Table 2). MDR was found in 90.80% of the tested isolates as illustrated in (Table 3).

**Table 2:** Antimicrobial resistance of NTS isolates (n = 87)

	Isolates of animal origin				Isolates of human origin		Total	
Antimicrobial agents	Milk and milk product isolates (n = 41)		Fecal swabs (n = 26)		Stool and hand swabs (n = 20)		n = 87	
	No	%	No	%	No	%	No	%
Streptomycin (S)	40	97.56	26	100	20	100	86	98.85
Clindamycin (CL)	41	100	26	100	20	100	87	100
Nalidixic acid (NA)	35	85.37	25	96.15	19	95	79	90.80
Penicillin G (P)	30	73.17	20	76.92	16	80	66	75.86
Norocillin (NO)	25	60.98	17	65.38	14	70	56	64.37
Tetracycline (T)	21	51.22	14	53.85	11	55	46	52.87
Sulphamethoxazol (SXT)	17	41.46	6	23.08	10	50	33	37.93
Kanamycin (K)	13	31.71	5	19.23	7	35	25	28.74
Ampicillin (AM)	10	24.39	3	11.54	7	35	20	22.99
Doxycycline (DO)	7	17.07	3	11.54	7	35	17	19.54
Ciprofloxacin (CP)	5	12.20	3	11.54	5	25	13	14.94
Gentamicin (G)	3	7.32	3	11.54	1	5	7	8.05
Cephalothin (CN)	3	7.32	1	3.85	1	5	5	5.75
Amikacin (AK)	1	2.44	0	0	1	5	2	2.30

**Table 3:** MDR of different NTS serotypes

Serotype	Total no	No. of MDR isolates	%
S. typhimurium	21	21	100
S. enteritidis	18	17	94.44
S. tsieve	11	11	100
S. infantis	7	6	85.71
S. heidelberg	2	2	100
S. larochelle	8	6	75
S. virchow	5	4	80
S. molade	7	6	85.71
S.haifa	3	3	100
S. essen	2	1	50
S. shubra	1	1	100
S. alfort	1	1	100
S. apeyeme	1	0	00.00
Total	87	79	90.80

Out of the 79 MDR NTS isolates, 36 isolates were tested by PCR for the presence of virulence genes including *invA*, *stn*, *spvC* and *hilA* genes. The occurrence of *invA* gene was 100% while, 72.22%, 30.56% and 94.44% of the examined stains were positive for *stn*, *spvC* and *hilA* genes, respectively (Table 4).

**Table 4:** occurrence of virulence genes of some NTS isolates (n = 36 strains)

Virulence genes *S.* Serovars	No. of tested strains	*invA*		*stn*		*spvC*		*hilA*	
		No	%	No	%	No	%	No	%
S. enteritidis	4	4	100	3	75	4	100	4	100
S. typhimurium	4	4	100	4	100	1	25	4	100
S. infantis	4	4	100	4	100	1	25	4	100
S. tsevie	4	4	100	0	0	0	0	4	100
S. larochelle	4	4	100	3	75	3	75	4	100
S. virchow	3	3	100	3	100	0	0	3	100
S. Haifa	3	3	100	3	100	0	0	3	100
S. molade	3	3	100	2	66.7	1	33.3	3	100
S. heidelberg	2	2	100	1	50	0	0	1	50
S. essen	2	2	100	1	50	0	0	2	100
S. shubra	1	1	100	1	100	1	100	1	100
S. alfort	1	1	100	1	100	0	0	0	0
S. apeyeme	1	1	100	0	0	0	0	1	100
Total	36	36	100	26	72.22	11	30.56	34	94.44

The same 36 isolates were investigated for the presence of β-lactamase antibiotic resistance genes including *blaOXA-2* and *blaCMY-1* genes. The *blaOXA-2* was detected in 16.67% (6 out of 36) of the tested isolates while, the *blaCMY-1* was detected in 30.56% (11out of 36) of the tested isolates (Table 5).

**Table 5:** occurrence of β-lactamase antibiotic resistance genes in some NTS isolates (n = 36 strains)

Virulence genesS. serovars	No. of tested strains	*blaOXA-2*		*blaCMY-1*	
		No	%	No	%
S. enteritidis	4	0	0	0	0
S. typhimurium	4	1	25	1	25
S. infantis	4	1	25	2	50
S. tsevie	4	0	0	1	25
S. larochelle	4	1	25	1	25
S. virchow	3	1	33.3	3	100
S. haifa	3	0	0	2	66.7
S. molade	3	1	33.3	0	0
S. heidelberg	2	0	0	0	0
S. essen	2	0	0	1	33.3
S. shubra	1	1	100	0	0
S. alfort	1	0	0	0	0
S. apeyeme	1	0	0	0	0
Total	36	6	16.67	11	30.56

### Phylogenetic analysis

Collectively, sequencing and phylogenesis of two *Salmonella* isolates, with GenBank accession number op973126 and op973127, (one from animal and one from human) which based on *invA* gene in this study revealed high degree of similarity between the two isolates and also, between the two isolates and those retrieved from the GeneBank.

## Discussion

Non-typhoidal *Salmonella* typically causes mild self-limiting gastroenteritis in the majority of people. The illness can also present as a febrile invasive disease, often without diarrhoea, with bacteraemia, meningitis, or focal infections that can be fatal if left untreated or improperly treated [17].

As illustrated in (Tables 1 and 6), the overall prevalence of NTS was 8.3% and 11.96% in humans and animals, respectively. The isolates recovered from all examined samples (87) were serotyped into 13 different serotypes, with differences in their distribution in different sample types. *S. typhimurium* and *S. enteritidis* were the predominant serotypes in animal and human isolates.

**Table 6:** Occurrence of different serotypes of NTS in different positive isolates

*Sallmonella* serotype	No. of isolates	%
Milk and milk products (n = 41)		
S. typhimurium	9	21.95
S. enteritidis	8	19.51
S. tsieve	6	14.63
S. infantis	5	12.20
S. larochelle	1	2.44
S. virchow	2	4.88
S. molade	3	7.32
S. haifa	2	4.88
S. shubra	1	2.44
S. alfort	1	2.44
S. essen	1	2.44
S. apeyeme	1	2.44
S. heidelberg	1	2.44
Fecal isolates (n = 26)		
S. typhimurium	8	30.77
S. enteritidis	5	19.23
S. tsieve	2	7.69
S. infantis	1	3.85
S. larochelle	4	15.38
S. virchow	3	11.54
S. molade	1	3.85
S. haifa	1	3.85
S. essen	1	3.85
Human isolates (n = 20)		
S. typhimurium	4	20
S. enteritidis	5	25
S. tsieve	3	15
S. infantis	1	5
S. larochelle	3	15
S. molade	3	15
S. heidelberg	1	5

Many authors in previous studies agree with us in the opinion that the most common serotypes of *salmonella* in humans and animals were *S. typhimurium* and *S. enteritidis* [15, 18–20]. However, a previous study in Ethiopia demonstrated that the most common serovar were S. dublin and S. Virchow [21]. Additionally, S. newport and S. typhimurium were the most predominant in Colombia by (60.87%) and (17.4%) respectively [22].

**Fig. 1:**
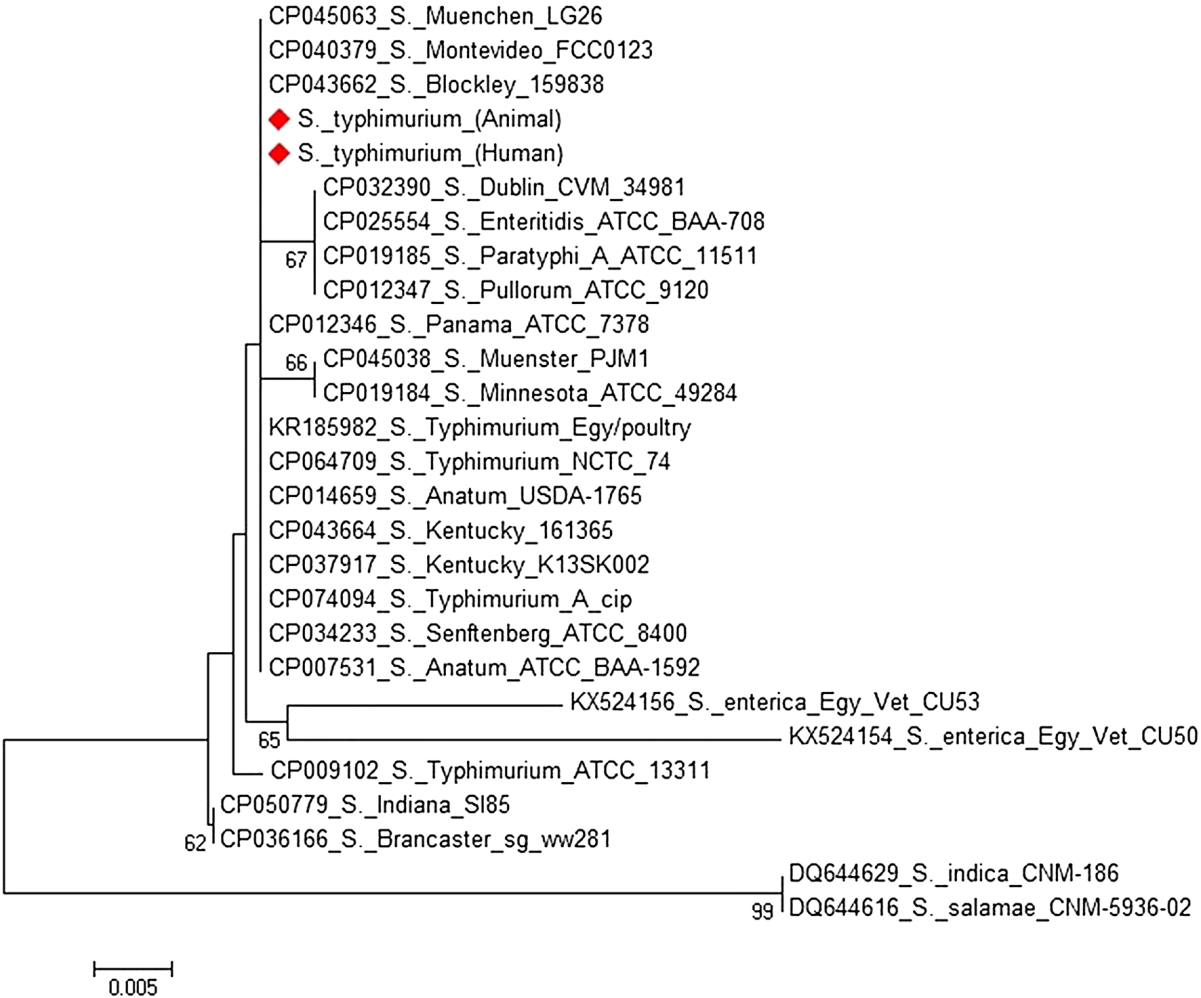
Phylogenetic tree of two *S. typhimurium* isolated from human and animal based on *invA* gene sequencing

NTS is the most common cause of acute gastroenteritis in humans. Antibiotic resistance has become a global issue as antibiotic use has increased [23]. Antimicrobial resistance refers to the ability of a microorganism to survive and reproduce in the presence of previously effective antibiotic doses. The results of antibiotic susceptibility testing are illustrated in (Table 2); the recovered NTS isolates showed 100% resistance to clindamycin, followed by streptomycin (98.85). These results are consistent with those obtained by many previous studies [15, 24] and It was observed that 100% of isolates from both animals and humans were resistant to clindamycin, although its use in large animals was limited. This demonstrated the cross-transmission of NTS between humans and animals (Fig. 1).

The high levels of resistance observed in the present study may be due to the indiscriminate use of drugs for the treatment of both human and animal diseases as a result of self-administration of drugs without proper clinical examination and cessation of drug usage before the complete dose. There was also poor knowledge of withdrawal time of used drugs, although different antibiotic classes of drugs are used in animal health management and in human medicine, the selection of resistance to one drug class may lead to cross-resistance to another [13, 23, 25, 26].

In contrast, amikacin showed the highest level of NTS susceptibility, followed by cephalothin, gentamicin, and ciprofloxacin. Similarly, the NTs isolates that were recovered from retail food in Thailand remain sensitive to amikacin [14].

The global incidence of MDR in *Salmonella* has increased over the last few decades [27]. Infection of humans with MDR strains of *Salmonella* has been reported to be associated with an increased burden of morbidity, extended hospitalization, increased risk of invasive illness, and increased mortality compared to those infected with susceptible strains [28]. As shown in (Table 3), MDR was found in 90.80% of the tested isolates. 100% of S. typhimuruim, S. tsieve, S. heidelberg, S. Haifa, S. shubra, and S. alfort showed MDR, whereas 94.44%, 85.71%, 75%, 80%, 85.71%, and 50% of S. enteritidis, S. infantis, S. larochelle, S. Virchow, S. molade, and S. essen, respectively, showed MDR against the antibiotics used. Lower occurrence of MDR were observed among the tested *Salmonella* isolates by 70, 13.2, 50, and 47.06% [29–31]. The differences in MDR between studies may be due to different types of samples, different types of antibiotics tested, different strains of *Salmonella*, and the frequent development of resistant genes.

The virulence of *Salmonella* is linked to a combination of chromosomal and plasmid factors. Of the 87 *Salmonella*-positive isolates, 36 isolates exhibiting MDR were tested by PCR for the presence of virulence genes, including *invA, stn, spvC, and hilA*. The occurrence of the *invA* gene was 100%, and 72.22%, 30.56%, and 94.44% of the examined strains were positive for *stn, spvC*, and *hilA* genes, respectively (Table 4).

The *invA* gene is considered a universal genetic marker identified in most *Salmonella* serovars [32]. Our results confirmed 100% occurrence of *invA* gene in the examined isolates, as recorded in many previous studies [^15^, ^33^, ^34^, ^35^]. However, only 67% of the examined samples were *invA* gene positive [5].

Data in (Table 4) illustrated that *stn* gene was found in 72.22% of the tested isolates. Similarly, the *stn* genes were detected in 71.7% of the tested isolates in Egypt [24]. On the other hand, the *stn* gene could be detected in all *Salmonella* isolates [34, 36, 37]. In contrast,

Of the 36 NTS isolates tested for the presence of virulence genes, 30.56% (11) were carrying the *spvC* gene (Table 4). Slightly higher percentages than ours were reported in Zambia and Burkina Faso by 24% and 58.8% respectively, [5, 38]. On the other hand the *spvC* gene could be detected in 100% of examined isolates in Egypt and Bangladesh [34, 36].

As shown in (Table 4)*, hilA* were detected in 94.44% of the examined isolates. The presence of the *hilA* virulence gene in the examined NTS serovars was detected by lower percentage 66.6% [39]. Moreover, the *hilA* gene was detected in all examined NTS isolates [35, 40].β-Lactam antibiotics are widely used in many developing countries. This group of antibiotics belongs to a family of antibiotics with a β-lactam ring, such as penicillin and cephalosporins. Members of β-lactam antibiotics are inactivated by bacteria through the production of β-lactamases, which hydrolyze the β-lactam rings. Many genes such as *blaCMY-1* and *blaOXA-2* are responsible for this mechanism. The isolates were tested for the presence of these two β-lactamase antibiotic resistance genes by PCR. *blaOXA-2* was detected in 16.67% (6 36) of the tested isolates, whereas *blaCMY-1* was detected in 30.56% (11out 36) of the tested isolates (Table 5).

These genes were detected in Egypt with lower incidence rates where they were *blaCMY-1* 10.5%, followed by *blaOXA-2* 6.6% [41]. On the other hand, other authors failed to detect *blaCMY-1* and blaCMY-2 in *Salmonella* isolates [^42^ and ^43^].

Collectively, sequencing and phylogenesis of two *Salmonella* isolates (one from animals and one from humans), which was based on the *invA* gene in this study as shown in Fig. 1, revealed a high degree of similarity between the two isolates, and those retrieved from GenBank. This finding was compatible with the results obtained by other authors and demonstrates the zoonotic cycling of *Salmonella* between animals and humans [44, 45]

## Conclusion

There is a high prevalence of antibiotic-resistant NTS in the feces of cows, milk and its products, as well as in human stool. Genetic analysis showed a very high rate of convergence between both isolates, which indicates the danger of transmission of NTS between humans and animals, determines the possibility of NTS transmission to human via milk and milk products with difficulty in treatment, which reflects its significant impact on public health.

## Materials

### Ethical declaration

This research was conducted in accordance with the guidelines of the Institutional Animal Care and Use Committee of New Valley University in Egypt. Informed consent was obtained from human participants and/or their legal guardians after receiving detailed information about the aims of the study.

### Study area and design

Samples were collected from different localities in the New Valley and Assiut Governorates, Egypt, between April 2020 and May 2021.

### Sampling

to isolate *Salmonella*, from April 2020 to May 2021 from different localities in New Valley and Assiut Governorates, Egypt, 800 samples, including raw milk (80), milk products (kareish cheese (80), Damietta cheese (80), yoghurt (80), ice cream (80), animal fecal swabs (160), human stool swabs (170), and hand swabs (70) were collected aseptically in sterile and sealed containers, labeled, placed in an ice box at 4 °C, and transported to the laboratory for microbiological analysis.

### Microbiological analysis

*Salmonella* was detected using conventional culture-based methods according to **ISO** [46]. 1 ml from the sample was inoculated in 9 ml of buffered peptone water (HiMedia M1494I) and incubated at 37 °C for 24 h. 1 ml of the homogenate was added aseptically to 9 ml of Rappaport Vassilliadis R10 medium (RV) (HiMedia M1530) and incubated overnight at 42 °C. A loopful from the enriched broth was inoculated onto xylose lysine desoxycholate (XLD) agar (HiMedia M031I)and then incubated at 37 °C for 24 h. Typical colonies of *salmonella* (pink to red with or without black center) were picked and streaked onto nutrient agar slopes and incubated at 37 °C for 18–24 h for biochemical identification.

Biochemical identification was performed according to MacFaddin [47] using the indole production test, Simmons citrate test, urease test, triple sugar iron, and sugar fermentation tests, and then confirmed using API (analytical profile index).

### Serotyping

The biochemically identified *Salmonella* isolates were serologically typed according to the Kauffman-White scheme [48] at the Faculty of Veterinary Medicine, Department of Food Hygiene and Control, Benha University, Egypt, using the slide agglutination technique for both somatic (O) and flagellar (H) antigens.

### Antibiotic resistance (Antibiogramme)

According to the CLSI [49] *Salmonella* isolates were tested for the antimicrobial susceptibility by the single diffusion method against 14 different antibiotics including nalidixic acid (30 µg), ciprofloxacin (5 µg), tetracycline (30 µg), penicillin G(10 IU), clindamycin (10 µg), norocillin (25 µg), cephalothin (30 µg), streptomycin (10 µg), doxycycline (30 µg), kanamycin (30 µg), ampicillin (10 µg), amikacin (30 µg), gentamicin (10 µg) and sulphamethoxazol (25 µg).

### Molecular detection of *Salmonella* virulence genes

Bacterial DNA was extracted Using GeneJET Genomic DNA Purification Kit (ThermoFisher, Cat. No. K0702 [50]. PCR was applied to amplify *salmonella* virulence genes *invA, hilA*, *stn* and *spvC* genes using specific primers as shown in (Table 7). PCR reaction mixture (25 µl) contained 2 µl of bacterial DNA, 2.5 µl of 10x Master Mix Green Master, Promega, USA) (containing 2.5 U of Taq DNA polymerase, 2.5 of 25 mM MgCl2 and 0.5 µl of 10mM dNTP mix), 0.5 µl of 1.2 µM primer mix (Applied Biosystem, USA) and 14.2 µl deionized water. The amplification done in Gradient Thermal Cycler (Veriti Applied Biosystem, USA). The PCR cycling protocol was applied as following: an initial denaturation at 94 °C for 2 min, followed by 30 cycles of denaturation at 94 °C for 45 sec, annealing at 53 °C for 1 min and extension at 72 °C for 1 min, followed by a final extension at 72 °C for 7 min. Finally, 5 µl of each amplicon was electrophoresed in 1.5 % agarose gel, stained with ethidium bromide and visualized and captured on UV transilluminator (UV, INC, UK). A 100 bp DNA ladder was used as a marker for PCR products.

**Table 7:** Primer sequences for certain virulence factors

Target gene	Oligonucleotide sequence (5′ → 3′)	Product size (bp)	References
*invA* (F)	5′ TATCGCCACGTTCGGCAA ′3	275	[51]
*invA* (R)	5′ TCGCACCGTCAAAGGAACC ′3		
*stn* (F)	5′TTGTGTCGCTATCACTGGCAACC ′3	617	[52]
*stn* (R)	5′ ATTCGTAACCCGCTCTCGTCC ′3		
*spvC* (F)	5′ CGGAAATACCATCAAATA ′3	669	[53]
*spvC* (R)	5′ CCCAAACCCATACTTACTCTG ′3		
*hilA* (F)	5′CGGAAGCTTATTTGCGCCATGCTGAGGTAG′3	854	[54]
*hilA* (R)	5′ GCATGGATCCCCGCCGGCGAGATTGTG ′3		

### Molecular detection of *Salmonella* B-lactamase resistance genes

PCR was applied to amplify *salmonella* virulence genes *blaCMY-1 and blaOXA-2* genes using specific primers as shown in (Table 8).

**Table 8:** Primer sequences for B-lactamase resistance genes

Target gene	Oligonucleotide sequence (5′ → 3′)	Product size (bp)	Reference
*blaCMY-1* (F)	5′ GTGGTGGATGCCAGCATCC ′3	915	[42]
*blaCMY-1* (R)	5′ GGTCGAGCCGGTCTTGTTGAA ′3		
*blaOXA-2* (F)	5′ ACGATAGTTGTGGCAGACGAAC ′3	602	
*blaOXA-2* (R)	5′ ATYCTGTTTGGCGTATCRATATTC ′3		

### Phylogenetic analysis

Sequencing and phylogenesis of two S. typhimurium isolates (one from animal and one from human) was performed using the *invA* gene. The *invA* gene sequences were aligned with CLUSTRAL W multiple sequence alignment program, version 1.83 of megalign module of lasergene DNAStar software pairwise, which was designed by [55] and phylogenetic analysis were done using maximum likelihood, neighbor joining and maximum parsimony in MEGA6 [56].

## Data Availability

The datasets used and/or analyzed in the current study were not publicly published to preserve the privacy of the participants but are available upon reasonable request from the corresponding author.
